# mGWAS identification of six novel single nucleotide polymorphism loci with strong correlation to gastric cancer

**DOI:** 10.1186/s40170-021-00269-2

**Published:** 2021-09-26

**Authors:** Shuangfeng Yang, Yuan-Liang Wang, Yanping Lyu, Yu Jiang, Jianjun Xiang, Shumi Ji, Shuling Kang, Xuejie Lyu, Chenzhou He, Peixin Li, Baoying Liu, Chuancheng Wu

**Affiliations:** 1 School of Public Health, Fujian Medical University, Fuzhou, China; 2 Fujian Provincial Key Laboratory of Environment Factors and Cancer, Fuzhou, China

**Keywords:** Metabolite, GWAS, Biomarkers, Gastric cancer

## Abstract

**Background:**

Metabolite genome-wide association studies (mGWAS) are key for understanding the genetic regulation of metabolites in complex diseases including cancers. Although mGWAS has revealed hundreds of metabolomics quantitative trait loci (mQTLs) in the general population, data relating to gastric cancer (GC) are still incomplete.

**Methods:**

We identified mQTLs associated with GC by analyzing genome-wide and metabolome-wide datasets generated from 233 GC patients and 233 healthy controls.

**Results:**

Twenty-two metabolites were statistically different between GC cases and healthy controls, and all of them were associated with the risk of gastric cancer. mGWAS analyses further revealed that 9 single nucleotide polymorphisms (SNPs) were significantly associated with 3 metabolites. Of these 9 SNPs, 6 loci were never reported in the previous mGWAS studies. Surprisingly, 4 of 9 SNPs were significantly enriched in genes involved in the T cell receptor signaling pathway.

**Conclusions:**

Our study unveiled several novel GC metabolite and genetic biomarkers, which may be implicated in the prevention and diagnosis of gastric cancer.

## Introduction

Gastric cancer (GC) is one of the most common, multifactorial malignancies mediated by environmental and genetic factors [1, 2]. Several genome-wide association studies (GWAS) have been performed to identify genetic biomarkers for GC [3–7]. Although multiple single nucleotide polymorphisms (SNPs) have been found to associate with GC, the effect size is small and the influence of genetic factors on the biological processes underlying GC remains elusive.

GWAS with intermediate phenotypes, like changes in metabolite and protein levels, are key in establishing functional links between genetic variants and disease end points [8]. Metabolomics is a rapidly developing discipline that focuses on the study and analysis of small endogenous molecules (MW < 1500 Da) [9]. As the endpoint of the signaling cascade, it represents the final response of living systems to environmental, genetic, and disease factors [9, 10]. Advances in metabonomic tools provide a unique opportunity to reveal complex relationships between genotypes and phenotypes [10]. Therefore, metabonomics may be an ideal intermediate phenotype choice. Prior studies have shown that GC is associated with alterations in circulating metabolites, such as nucleotides, lipids, and amino acids [11, 12]. Nevertheless, the relationship between metabolite levels and biological mechanisms of GC remains unclear.

GWAS for metabolic traits (metabolite genome-wide association studies, mGWAS) has revealed hundreds of metabolomic quantitative trait loci (mQTLs) in the general population [10, 13–15]. For GC, the identification of novel mQTLs may provide a valuable tool for discovering genetic biomarkers related to GC and provide new insights from the perspective of metabolism. Mapping GC-associated mQTLs could also shed light on the etiology and mechanisms of GC, which in turn is important for the prevention, early detection, diagnosis, and targeted treatment of this malignancy. However, to our knowledge, there are currently no studies of MGWAS associated with GC.

To identify the GC-associated mQTLs for the first time, we combined genome-wide and metabolome-wide datasets generated from 233 GC patients and 233 healthy controls. Patients were age-, sex-, smoking status-, alcohol consumption-, *H. pylori* infection-, and time of blood sample collection-matched to healthy controls. We hypothesized that by combining phenotypic, metabolomic, and genetic data, we could better identify individuals at risk of GC and uncover biological mechanisms related to GC.

## Materials and methods

### Study samples

This study was based on a population-based case-control study, in which GC patients and healthy individuals were consecutively enrolled in Xianyou County between March 2013 and December 2017. All cases were newly diagnosed histologically based on tissue specimens and had lived in Xianyou for at least 10 years. GC patients with other cancers, secondary or recurrent gastric cancer, gastritis, previously received neoadjuvant chemotherapy or chemoradiotherapy or radiotherapy, pregnant, metabolic diseases such as diabetes, gout, hyperlipidemia, systemic administration of corticosteroids, neurological and psychiatric diseases, severe hepatic and renal dysfunction, and severe respiratory disease requiring continuous oxygen treatment, etc., were excluded. For the healthy individuals, there was no abnormality of blood tests and endoscopic examinations.

The study initially included 244 GC cases and 244 healthy individuals. Ethical approval for the study was obtained from the Biomedical Research Ethics Committee of Fujian Medical University, China (No. 97,2014). Following an explanation of the study, written informed consent was obtained from all participants at study enrollment.

### Metabolite profiling

Blood samples were collected after an overnight fast, immediately centrifuged, and stored at −80°C until assayed. Plasma metabolites were profiled as previously described using Agilent 1200 high-performance liquid chromatography combined with a 6520 accurate electrospray ionization/quadrupole-time-of-flight mass system (Agilent Technologies, CA, USA) [16]. A total of 225 compounds were detected. If a variable had a nonzero measurement value in at least 80% of the variables within one of the two subsets, the variable was included in the data set; otherwise, the variable was removed. This procedure will be referred to as the “80% rule” [17]. After being excluded based on extracted ion chromatogram (EIC) and “80% rule,” 60 metabolites have remained for subsequent analyses. The final metabolomics dataset contained 20 nucleotides, 19 lipids, 7 amino acids, 8 organoheterocyclic compounds/others, 3 peptides, and 3 unknown.

### Genome-wide genotyping

Approximately 900,000 SNPs were genotyped using the Axiom™ Precision Medicine Research Array (Thermo Fisher Scientific, Waltham, MA, USA). Genotyping was performed as described according to the manufacturer’s instructions. Briefly, genomic DNA was extracted using a Genomic DNA Isolation Kit (Biovision, CA, USA). Each sample was whole-genome amplified, fragmented, precipitated, dried, resuspended in appropriate hybridization buffer, chip cleaned, stained, and scanned. Participants were excluded if they satisfy any of the following items: (1) low call rate (overall rate < 95%), (2) ambiguous gender, and (3) duplicates or familial relationship (PI_HAT > 0.025). SNPs were excluded if they (1) were not mapped to autosome chromosomes, (2) had a call rate < 95%, (3) had minor allele frequency (MAF) < 0.05 in controls, and (4) were an excessive deviation from Hardy-Weinberg equilibrium in controls (*p* < 1 × 10^−6^). As a result, 233 pairs of subjects and 258,544 SNPs were left for subsequent analysis.

### Statistical analysis

Before statistical analysis, each metabolic peak in all subject samples was normalized based on QC samples for removing the unwanted analytical variations occurring intra- and inter-batches. And the plasma abundant values of metabolites investigated were set to a log scale and auto-scaled (mean-centered and divided by the standard deviation of each variable) using MetaboAnalyst 4.0. An orthogonal partial least squares discriminant analysis (OPLS-DA) and univariate two-sides *t*-test were used for metabolic profile description and metabolic signature discovery between GC cases and healthy controls. The false discovery rate (FDR) method was used to correct for multiple hypothesis testing and to reduce false positives. Those metabolic features with variable importance in the projection (VIP) values > 1.0 in the OPLS-DA model and FDR-adjusted *p* values < 0.05 in the *t*-test were considered to be significantly different between GC cases and healthy controls. Then, logistic regression was performed to test the association between discriminant metabolites and incident GC.

After that, we analyzed the associations between discriminant metabolite levels and genome chip variants using a generalized linear model adjusted for age, sex, family relationship, smoking status, alcohol consumption, pickled vegetable intake, and *H. pylori* infection analysis in TASSEL software (version 5.0). To control for false-positive error rates deriving from the large number of SNPs tested, a conservative Bonferroni-adjusted *p*-value of *p* = 8.79 × 10^−9^ (= 0.05/(258544SNPs × 22 metabolites)) was applied for declaring genome-wide significance for the SNP-metabolite associations. The significant SNPs were annotated to the neighboring genes of 1000 Genomes Project (hg19/1000 Genomes ASN) downloaded from the University of California Santa Cruz (UCSC) genome browser (http://hgdownload.cse.ucsc.edu). Then, we conducted Gene Ontology (GO) enrichment analysis to investigate possible biological, molecular, or cellular processes associated with significant SNP-related genes using Metascape (https://metascape.org/) [18]. Furthermore, the interaction network between metabolites, genes, and GO terms were visualized using Cytoscape [19]. Finally, RNA-seq data of gastric cancer tissue and adjacent normal tissue of six gastric cancer patients in Asia were downloaded from The Cancer Genome Atlas (TCGA; https://www.cancer.gov/tcga), and *t* test was performed to compare the candidate gene expression between two different groups shown in boxplot using R (R version 3.6.0).

## Results

### Study sample characteristics

A total of 233 GC cases and 233 healthy controls were included, with the mean age of 64.80 ± 7.89 years and 65.16 ± 7.91 years, respectively. As shown in Table 1, except for pickled vegetable intake (*p* < 0.05), there was no statistically significant difference in gender, age, smoking status, alcohol consumption, and *H. pylori* infection, between GC cases and healthy controls (*p* > 0.05).

**Table 1:** Demographic characteristics of study participants

	Control (***n*** = 233)	Case (***n*** = 233)	***χ***^**2**^	***p***-value
**Gender**			**0.000**	**1.00**
Female	63	63		
Male	170	170		
**Age**			**0.424**	**0.515**
≤65y	123	130		
>65y	110	103		
**Smoking status**			**0.009**	**0.926**
No	109	108		
Yes	124	125		
**Alcohol consumption**			**1.378**	**0.240**
No	194	203		
Yes	39	30		
**Pickled vegetable intake**			**17.003**	**<0.001**
No	66	30		
Yes	167	203		
***H. pylori*infection**			**0.567**	**0.451**
No	141	133		
Yes	92	100		

### Metabolic profiles and discriminant metabolites between GC cases and healthy controls

**Fig. 1:**
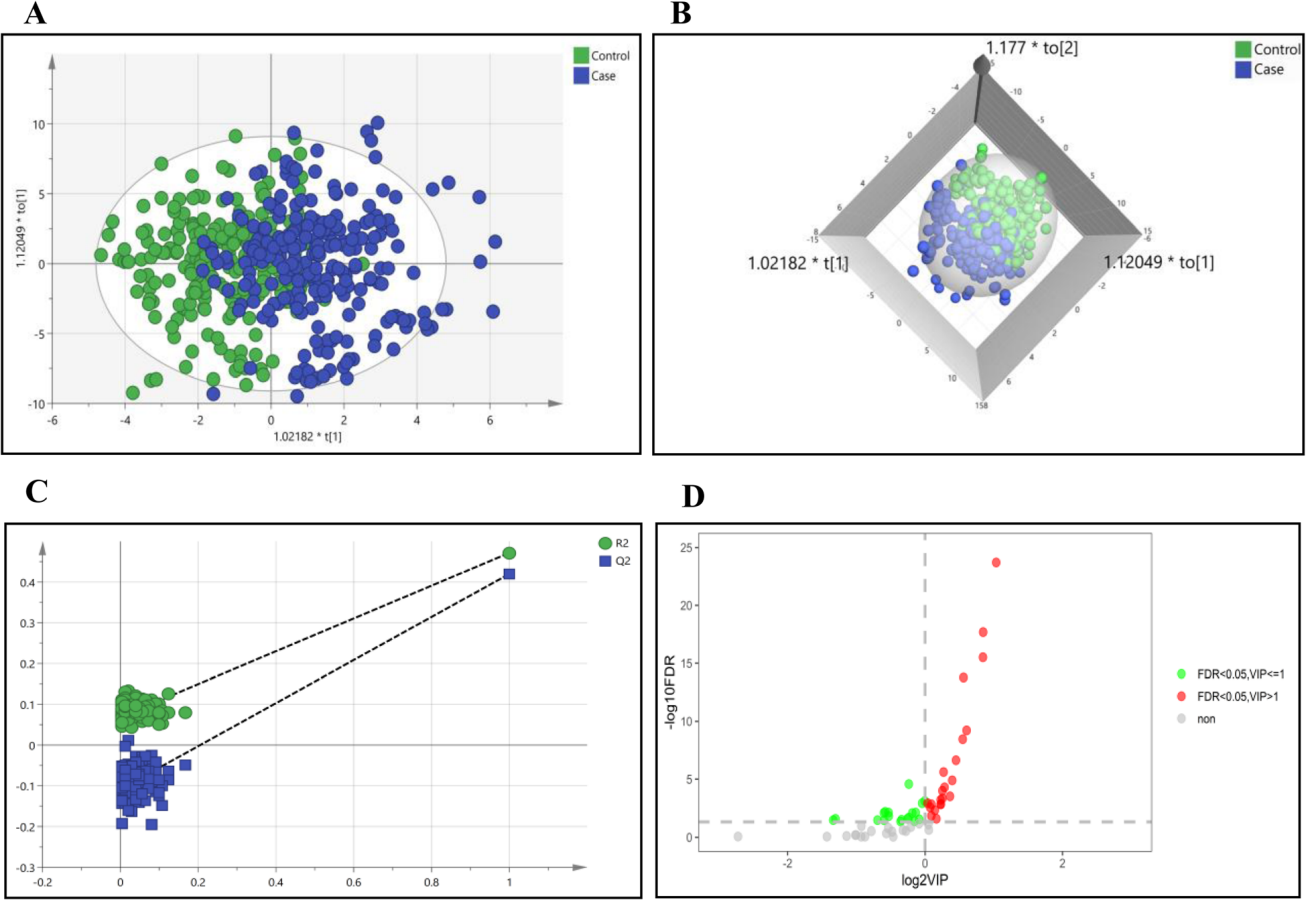
Metabolic profiles and discriminant metabolites between GC cases and healthy controls. **A** Orthogonal partial least-squares discrimination analysis (OPLS-DA) score plots. **B** OPLS-DA three-dimensional score plots. **C** Validation plots obtained from 200 permutation tests for the OPLS-DA models. **D** Volcano plot of discriminant metabolites between GC cases and healthy controls. Notes: blue, GC cases; green, healthy controls; red, discriminant metabolites

To identify potential compounds that could be used to differentiate the metabolite profiles of GC cases and healthy controls, we established a supervised OPLS-DA model that focused on the actual class discriminating variation. As shown in Fig. 1A and B, we observed a clear separation between the two groups. Response permutation testing was performed to verify the reliability of the OPLS-DA model. As shown in Fig. 1C, the goodness of fit (*R*^2^*Y*) and prediction ability of the model (*Q*^2^) were 0.472 and 0.420 for differentiating GC cases and healthy controls, respectively, suggesting successful model construction.

Subsequently, to find discriminant metabolites between GC cases and healthy controls, a univariate two-sides *t*-test and the VIP scores from OPLS-DA models were carried out. Metabolites with FDR-adjusted *p*-values < 0.05 and VIP >1 were selected as discriminants. Figure 1D shows that 22 metabolites were statistically different between GC cases and healthy controls, including 7 nucleotides, 9 lipids, and 6 others.

### Association between discriminant metabolites and gastric cancer

**Fig. 2:**
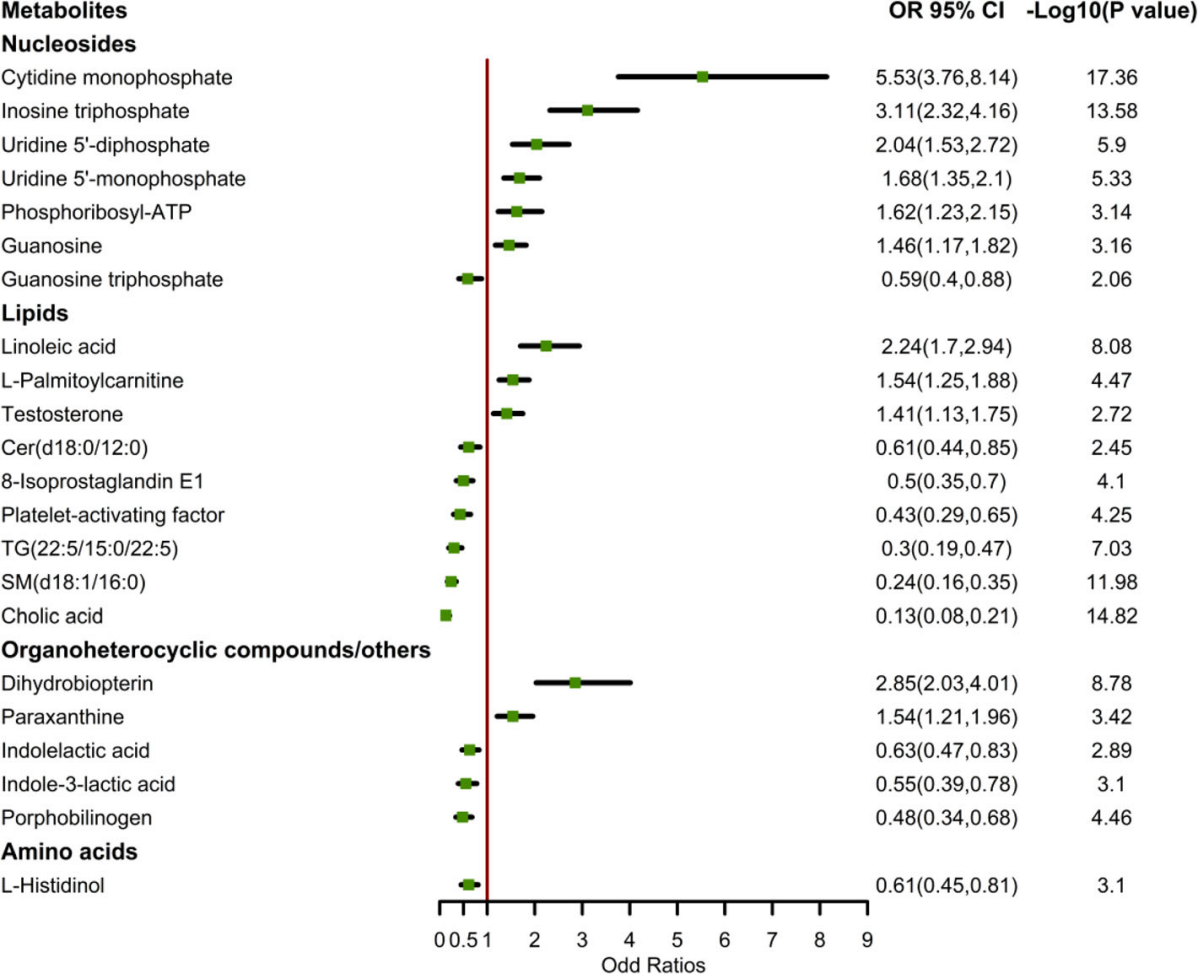
Odds ratios (OR) and 95% confidence intervals (CI) for discriminant metabolites and the risk of gastric cancer. Notes: OR is from logistic regression models adjusted for pickled vegetable intake status

Figure 2 denotes odds ratios for the association between 22 discriminant metabolites and GC risk, which was adjusted for pickled vegetable intake status. Eleven metabolites, including cytidine monophosphate (CMP), inosine triphosphate (ITP), uridine 5′-monophosphate (5′-UMP), uridine 5′-diphosphate (5′-UDP), guanosine, phosphoribosyl-ATP, linoleic acid, l-palmitoylcarnitine, testosterone, dihydrobiopterin, and paraxanthine, were associated with increased risks of GC. Conversely, metabolites including guanosine triphosphate (GTP), Cer(d18:0/12:0), 8-Isoprostaglandin E1, platelet-activating factor, TG(22:5/15:0/22:5), SM(d18:1/16:0), cholic acid, indolelactic acid, indole-3-lactic acid, porphobilinogen, and l-histidinol were associated with decreased risk of GC. Receiver operating characteristic (ROC) curve analysis was used to assess the discriminative ability of the 22 metabolites. The area under the curve was 0.897 (*95% CI* 0.869 to 0.924), indicating a high diagnostic value for GC. In short, these metabolites are highly correlated with the occurrence of GC.

### Genetic associations of discriminant metabolites

Next, we analyzed the association of these 22 metabolites with genome chip variants. As listed in Table 2, 9 SNPs were significantly associated with 3 metabolite concentrations at a stringent genome-wide cutoff of 8.79 × 10^−9^. Of these, 4 loci are in the enhancer regions, 2 are in the 3′UTR. Besides, 6 loci have not been reported before, while 3 loci were reported in the previous mGWAS study in the general population [20], including rs12204145, rs12208390, and rs3927423. The last two loci were in strong linkage disequilibrium (LD) (*r*^2^ = 1) with the variant rs12202419. And markers *R*^2^ for 3 metabolites ranged from 7.23 to 8.46% and the total phenotypic variance explained by 9 SNPs was 70.46%.

**Table 2:** SNP associations of discriminant metabolites with *p* < 8.79 × 10^−9^

Metabolites	SNP	Chr	Pos	Alt	Ref	*p value*	*R*^2^	Candidate gene	Function
Dihydrobiopterin	rs140991639	2	226294958	A	G	6.62E−09	7.23%	NYAP2	Intron, Enh60914
Platelet-activating factor	rs12208390	6	26437106	T	C	1.69E−09	8.10%	BTN3A3,BTN2A3P	Upstream
	rs3927423	6	26393487	T	C	2.73E−09	7.93%	BTN2A2	UTR-3
	rs6912853	6	26401438	T	C	3.80E−09	7.82%	BTN3A1	Upstream
	rs140044870	4	150707364	A	C	5.88E−09	7.62%	DCLK2	Upstream, Enh90264
	rs12204145	6	26600156	A	G	7.73E−09	7.51%	ABT1	UTR-3
	rs957788	13	108257220	C	T	8.66E−09	7.51%	FAM155A	Intron
Cer(d18:0/12:0)	rs1183416	13	44873710	T	C	1.10E−09	8.46%	SERP2	Upstream, Enh30302
	rs295977	5	58893286	A	C	1.70E−09	8.28%	PDE4D	Intron, Enh55272

*SNP*, single nucleotide polymorphism; *Chr*, chromosome; *Pos*, position; *Alt*, alternative allele; *Ref*, reference allele

### Gene and metabolite interaction network

**Fig. 3:**
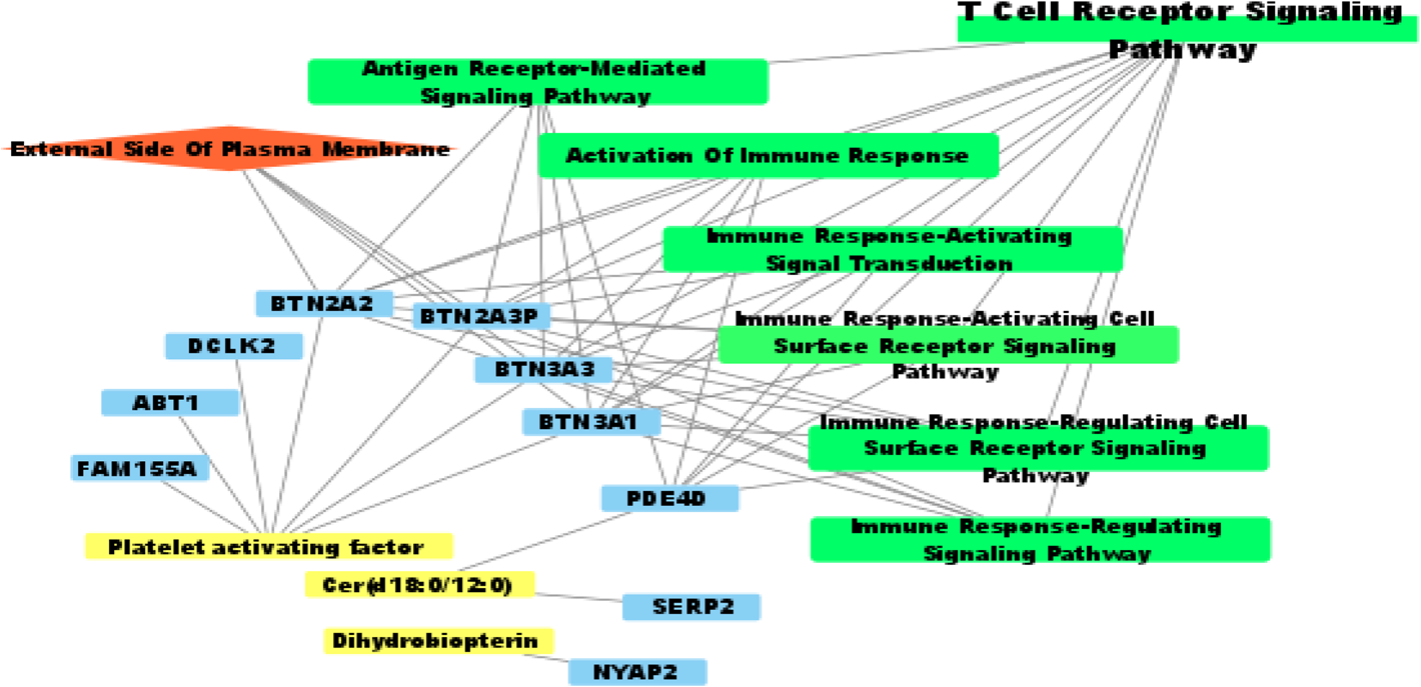
A network model describing the metabolites, genes, and GO term interactions. Notes: yellow rectangle, metabolites; blue rectangle, neighboring genes of significant SNPs; red diamond, GO cellular components; green rectangle, GO biological processes

To investigate which biological pathways were associated with these SNPs and the corresponding metabolites, we carried out a GO enrichment analysis. The most significantly enriched gene-based pathways were the T cell receptor signaling pathway (GO:0002376) with five genes (i.e., *PDE4D*, *BTN3A3*, *BTN2A2*, *BTN3A1*, *BTN2A3P*) (Fig. 3). Figure 3 also denotes the relationships between metabolites, genes, and GO terms.

### Expression of 10 candidate genes between gastric cancer tissue and adjacent normal tissue

**Fig. 4:**
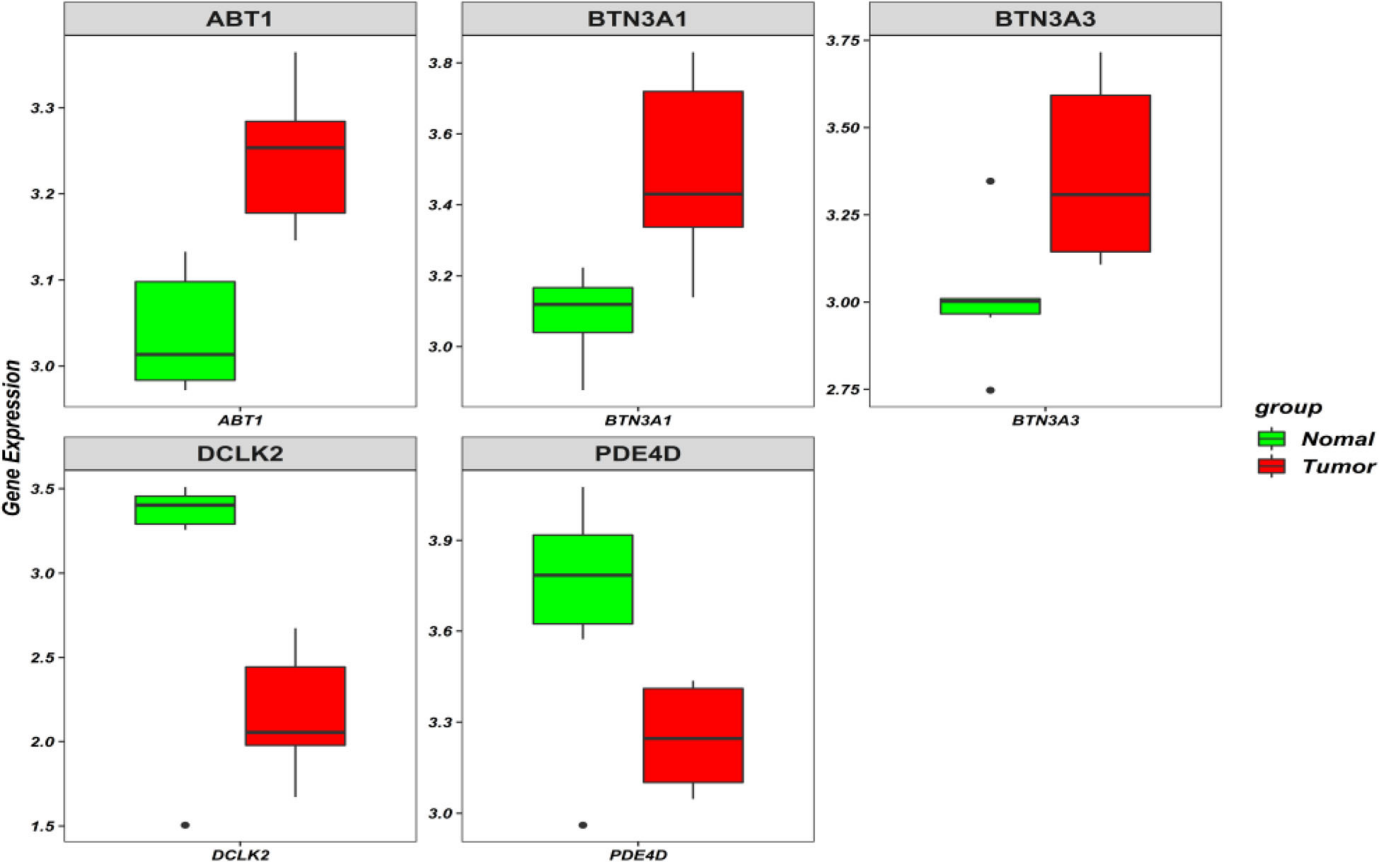
Differentially expressed genes between gastric cancer tissue and adjacent normal tissue among 10 candidate genes (*FDR_P value* < 0.05). Notes: green, adjacent normal tissue; red, gastric cancer tissue

Finally, GWAS was performed by plink to investigate whether the genotype distribution of the 9 SNPs is different between GC cases and healthy controls. Unfortunately, no statistically significant association was found. Then, we downloaded RNA-seq data of gastric cancer tissue and adjacent normal tissue from TCGA and compare the expression of 10 candidate genes between GC tissue and adjacent normal tissue. As shown in Fig. 4, there were statistically significant differences in the expression of 5 genes between the two groups (*FDR_P value* < 0.05), which indirectly verifies the reliability of this study.

## Discussion

### Discriminant metabolites between GC cases and healthy controls

In this study, 22 metabolites were found to be associated with the occurrence of GC, including 7 nucleotides, 9 lipids, 5 organoheterocyclic compounds, and 1 amino acid.

Nucleotide synthesis is key for cell proliferation as it is required for DNA replication, gene transcription, and ribosome biogenesis [21]. Tumor cells are in a state of such rapid proliferation and differentiation that frequent nucleotide synthesis and metabolism are upregulated significantly. Our study found that the relative abundance of CMP, 5′-UMP, 5′-UDP, ITP, guanosine, and phosphoribosyl-ATP were increased in the GC cases compared with healthy controls, while GTP was decreased. Previous research showed that guanosine was also increased in GC samples, but no noticeable difference of ATP and GTP was observed between normal and GC tissues [22]. This discrepancy may be due to the difference in experimental materials. Nucleotides in the blood are either passively released from stressed or dying cells due to cell lysis or actively released through membrane channels by exocytosis of vesicles or as part of exosomes [23]. The increase of nucleotides in plasma in our study may indicate an increase of dead cells in the blood of GC patients. On the one hand, it may come from the death of normal cells due to undernutrition [24, 25]. Alternatively, it may come from the increase of programmed cell death process triggered by cellular stress, DNA damage, and immune surveillance, to resist tumor proliferation [26].

Lipids, including phospholipids, fatty acids, triglycerides, sphingolipids, cholesterol, and cholesteryl esters, are key constituents of all biological membrane structures [27, 28]. Moreover, lipids could function as second messengers to transduce signals within cells, and serve as important energy sources when nutrients are limited [29]. As a result, proliferating cells need to acquire sufficient lipids to support membrane growth and integrity. Accumulating evidence has demonstrated that lipid metabolism is substantially reprogrammed in cancers including GC [11, 30]. In this study, linoleic acid, l-palmitoylcarnitine, testosterone, Cer(d18:0/12:0), 8-Isoprostaglandin E1, platelet-activating factor, TG (22:5/15:0/22:5), SM (d18:1/16:0), and cholic acid were found to be associated with GC. Yu et al. demonstrated that there was no difference in plasma linoleic acid concentration between gastric cancer patients and healthy individuals [31], but some studies also found that the increase of linoleic acid in plasma or diet may increase the risk of hepatocellular carcinoma, colorectal cancer, and prostate cancer [32, 33]. Linoleic acid can be metabolized to arachidonic acid, which plays an important role in inflammatory processes, as the latter can serve as a substrate for the production of some pro-inflammatory eicosanoids, leading to the production of inflammatory mediators such as tumor necrosis factor-alpha (TNF-α) and interleukin-1 (IL-1) [34]. Higher l-palmitoylcarnitine levels were found in mice with colon cancer and patients with acute pneumonia, indicating an increase in inflammation and fatty acid beta-oxidation [35, 36]. Findings of our study suggest that linoleic acid and l-palmitoylcarnitine may also play a pro-inflammatory role in gastric cancer.

Cer(d18:0/12:0) and SM(d18:1/16:0) are members of sphingolipids (SPs), which are structural molecules of cell membranes with important roles in maintaining barrier function and fluidity [35, 37]. Sphingolipids also regulate various biological processes such as growth, proliferation, migration, invasion, and/or metastasis by controlling signaling functions within the cancer cell signal transduction network [38]. Changes in cellular ceramide levels are followed by the activation of downstream effectors, which results in cell cycle arrest, senescence, or programmed cell death [39]. In this study, the decrease of Cer(d18:0/12:0) in plasma may indicate the disorder of the cell cycle and programmed cell death process, resulting in the exuberant proliferation of cancer cells and the continuous production of the cell membrane.

Platelet-activating factor, also known as PAF or PC(O-16:0/2:0), is a ubiquitous, potent phospholipid activator and mediator of inflammation that plays an important role in the pathogenesis of inflammatory disorders [40]. In vitro and animal studies suggest that PAF can act on the growth of various human tumor cell lines, increasing the adhesiveness of tumor cells to vascular endothelia, enhancing oncogene expression, and contributing to tumor development through enhancement of cell motility and stimulation of angiogenic response [41–44]. PAF receptor expression is increased in patients with gastric adenocarcinoma, and it is closely related to tumor proliferation ability and tumor size [45], which indicates that gastric adenocarcinoma tissue needs to consume more PAF. Transcripts of PAF and PAF receptors were also significantly increased in hepatocellular carcinoma specimens compared with non-cancer specimens [46]. In conclusion, PAF can change local angiogenesis and cytokine networks and is essential to suppress the immune system and promote metastasis and tumor growth [47]. In this study, the decrease of the platelet-activating factor in plasma may indicate the increase of the platelet-activating factor in the tumor microenvironment. However, these hypotheses need to be effectively verified by subsequent experiments.

The relative abundance of dihydrobiopterin (BH2), the precursor of tetrahydrobiopterin (BH4) of folate biosynthesis, was increased in gastric cancer patients compared with the healthy controls. Tetrahydrobiopterin (BH4) is essential for the synthesis of nitric oxide (NO) [48], and it is recognized as an essential step in initiating neoplastic transformation [49]. A previous study demonstrated that the BH4:BH2 ratio is lower in tumor tissues. As a consequence, nitric oxide synthase activity generates more peroxynitrite and superoxide anion than nitric oxide, resulting in tumor growth and anti-apoptotic signaling [50].

### Genetic associations of discriminant metabolites

Although we found some metabolites associated with GC, the biological mechanisms remain unclear. Hence, it is essential to combine metabolomics with other -omics methods to get a more integrated understanding of gastric carcinogenesis. mGWAS is an excellent method in quantifying metabolic data and uncovering genetic variants affecting metabolite levels [13]. In this study, the area under the ROC of 22 discriminant metabolites was 0.897 (*95% CI* 1.12 to 1.62), indicative of an ideal intermediate phenotype choice of GC. Therefore, we proceeded to identify mQTLs associated with GC by testing the genetic associations of 22 discriminant metabolites with genome-wide data. We found that 9 SNPs were significantly associated with 3 metabolites. Of them, 3 loci were reported in the previous mGWAS [20], and 6 loci were novel. Surprisingly, 4 of these 9 SNPs were significantly enriched in five genes (i.e., PDE4D, BTN3A3, BTN2A2, BTN3A1, BTN2A3P) involved in the T cell receptor signaling pathway (GO:0002376).

Besides, 6 SNPs were found to be associated with PAF, which influences the occurrence of gastric cancer. They included rs12208390, rs3927423, and rs6912853 in butyrophilins (BTN) gene; rs140044870 in DCLK2; rs12204145 in ABT1; and rs957788 in FAM155A. The BTN gene belongs to the immunoglobulin superfamily. There are seven human BTN genes in three related phylogenetic subfamilies. BTN1 subfamily contains only the BTN1A1 gene, while BTN2 contains BTN2A1, BTN2A2, and BTN2A3 pseudogenes, and BTN3 contains BTN3A1, BTN3A2, and BTN3A3 genes [51]. Studies have shown that overexpression of BTN3A and dominant expression of BTN3A2 subtypes are closely related to the poor prognosis of pancreatic ductal adenocarcinoma and gastric cancer [52, 53]. Overexpression of the BTN3A2 gene is associated with increased proliferation and invasion of gastric cancer cells [52]. Furthermore, Sarterk et al. found that BTN2A2 is a co-inhibitory molecule that modulates T cell-mediated immune responses [54]. Also, Lebrero-Fernandezc et al. found that BTN2A2 gene expression was significantly increased in the tissues of colon cancer patients compared with healthy controls [55]. Importantly, we found that the relative abundance of PAF in the plasma of the gastric cancer group and the control group was different, and PAF-related genes were mainly enriched in the T cell receptor signaling pathway. Therefore, we speculate that PAF-related SNPs may regulate PAF metabolism by regulating the expression of gastric cancer BTN family genes, mediate the T cell receptor signaling pathway, promote angiogenesis, and promote the growth and metastasis of gastric cancer cells.

Previous studies revealed that the Neuronal tyrosine-phosphorylated Adaptor for The PI3-kinase (NYAPs) could activate the PI3K signaling pathway [56]. Moreover, activation of the PI3K signaling pathway promotes cell growth and survival and is a significant cancer development regulator [57]. Consistently, this study found that the rs140991639 loci in NYAP2 were associated with BH2, which may be related to the occurrence of gastric cancer. Other targets found in this study that may be related to the occurrence of gastric cancer include DCLK2 (associated with reduced survival in cancer patients [58]), ABT1 (encodes proteins needed by ribosomes and contains genetic modifiers responsible for promoting nerve cell survival [59]), and FAM155A (associated with tumor invasion phenotype and early distant metastasis in patients with surgically treated renal clear cell carcinoma [60]). It is worth noting that the rs957788 in FAM155A was found to be associated with anorexia nervosa in a GWAS study [61]. Furthermore, we found rs295977 in PDE4D and rs1183416 in SERP2 were related to Cer(d18:0/12:0). To our knowledge, both PDE4D (phosphodiesterase 4D) and SERP2 (stress-associated endoplasmic reticulum protein family member 2) are associated with tumors [62, 63]. Among them, PDE4D has been reported to act as a proliferation promoter in several different tumors [64–66], and mutations in SERP2 are associated with leukemia [67]. In our study, both the 2 loci were connected to Cer(d18:0/12:0), but further studies are needed to explore the specific relationship between the occurrence of gastric cancer.

## Conclusions

In summary, we found 22 metabolites that were statistically different between GC cases and healthy controls. All of them were associated with the risk of gastric cancer. The area under the ROC of 22 discriminant metabolites was 0.897 (*95% CI* 0.869 to 0.924), indicating a high diagnostic value for GC and an ideal intermediate phenotype choice of GC. More importantly, we identified 6 new mQTLs in GC cases that may be a valuable tool for discovering genetic biomarkers related to GC. Therefore, this paper provides new insights into the prevention, early detection, diagnosis, and targeted treatment of gastric cancer. However, a validation cohort and in vivo and in vitro experiments are required to confirm these findings and fully explore the implications of this study.

## Data Availability

The datasets used and/or analyzed during the current study are available from the corresponding authors on reasonable request.
